# Ferritin as an Effective Prognostic Factor and Potential Cancer Biomarker

**DOI:** 10.3390/cimb47010060

**Published:** 2025-01-16

**Authors:** Katarzyna Szymulewska-Konopko, Joanna Reszeć-Giełażyn, Monika Małeczek

**Affiliations:** Department of Medical Pathomorphology, Medical University of Bialystok, 15-089 Białystok, Poland; katarzyna.szymulewska-konopko@umb.edu.pl (K.S.-K.); monika.maleczek@umb.edu.pl (M.M.)

**Keywords:** ferritin, cancer, biomarker

## Abstract

Ferritin is found in all cells of the body, serving as a reservoir of iron and protecting against damage to the molecules that make up cellular structures. It has emerged as a biomarker not only for iron-related disorders but also for inflammatory diseases and conditions in which inflammation plays a key role, including cancer, neurodegeneration, and infection. Oxidative stress, which can cause cellular damage, is induced by reactive oxygen species generated during the Fenton reaction, activating signaling pathways associated with tumor growth and proliferation. This review primarily emphasizes basic studies on the identification and function of ferritin, its essential role in iron metabolism, its involvement in inflammatory diseases, and its potential as an important prognostic factor and biomarker for cancer detection.

## 1. Introduction

Iron is a metal that is essential to the functioning of almost every form of life. Its transitory nature is of particular importance. This is due to its redox properties, which make iron a functional element of many proteins and enzymes involved in key biochemical processes. The properties of iron make it necessary for life, but they also determine its toxic effects, which are related to oxygen metabolism [1]. Therefore, living organisms are protected against oxidative damage by proteins that bind iron. Iron ions circulating in the plasma are bound to transferrin, while in cells, they accumulate in the form of ferritin. Ferritin is integral to our health and daily functioning. As the principal iron storage protein in the body, ferritin not only indicates current iron reserves but also plays a significant role in the diagnosis and management of various diseases. This discussion will primarily concentrate on the identification and function of ferritin, its critical role in iron metabolism, the regulation of ferritin under hypoxic conditions, the involvement of ferroportin and hepcidin in enhanced iron sequestration, its associations with inflammatory diseases, and its potential as a vital prognostic factor and biomarker in cancer detection.

## 2. The Identification and Function of Ferritin

Ferritin is a protein that, due to its ability to bind iron atoms, accumulates iron in cells in a form that is safe for the body, constituting its reserve. It is a protein with a high molecular weight (450 kDa), giving it the ability to bind up to 4500 iron atoms and creating the so-called mineral core in which this element is stored in the form of hydroperoxide complexed with phosphate. Ferritin stores iron and is one of the most important proteins involved in iron metabolism. Two types of ferritin have been recognized—cytosolic ferritin and mitochondrial ferritin (FTMT). Cytosolic ferritin stores excess iron in the core of the shell and releases it through degradation when it is needed. Ferritin synthesis is regulated not only by the iron content in cells but also by cellular stress, such as oxidative stress or inflammation. FTMT is classified as a metal-binding protein that is found in the mitochondria. Once the protein is taken up by the mitochondria, it can be processed into mature protein, and functional ferritin shells can be assembled. Mitochondria, which perform numerous bioenergetic, biosynthetic, and regulatory functions, play a key role in iron metabolism. Extracellular iron is taken up by cells and transported to the mitochondria, where it is used to synthesize the cofactors necessary for enzyme function, such as iron–sulfur clusters and heme. Although the physiological role of FTMT is still not fully understood, recent studies have shed light on the function and regulation of FTMT. There is increasing evidence of the presence of ferritin, allowing for a deeper understanding of iron metabolism and its role in disease [2].

**Figure 1:**
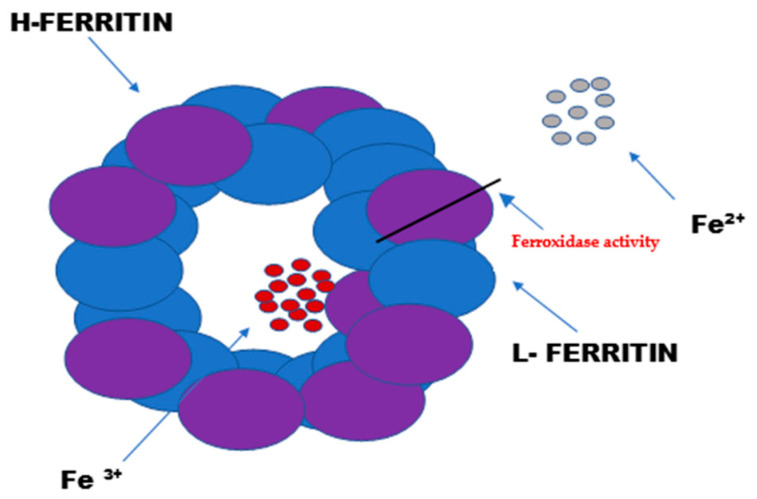
The structure and function of ferritin. Ferritin is the intracellular protein responsible for the sequestration, storage, and release of iron. Ferritin is a globular protein complex consisting of 24 protein subunits that form a hollow spherical nanocage with multiple metal–protein interactions. Ferritin with the iron removed is called apoferritin. Each apoferritin shell consists of 24 subunits of two types: the H subunit and the L subunit.

Ferritin consists of 24 subunits. Each of these subunits consists of four long helices twisted into pairs, thus creating the protein part of ferritin—apoferritin. The ferritin subunits consist of a light chain “L” (ferritin light chain) and a heavy chain “H” (ferritin heavy chain). The heavy chain (mass of 21 kDa) exhibits enzymatic activity (Figure 1). It acts as a ferroxidase by oxidizing iron ions from Fe^2+^ to Fe^3+^. The light chain (mass of 19 kDa) serves as the main iron storage facility. Depending on the location of the cells and their physiological state, the heavy-to-light-chain mass ratio may vary.

The L form of ferritin occurs mainly in the liver and spleen, while the H form predominates in the heart and kidneys [2,3,4]. The ferritin accumulated in serum is only a small fraction of the cellular ferritin, but it reflects the body’s iron stores under different physiological states [5,6].

Serum ferritin (SF) has a low iron content compared to its cellular form, but it may still play a major role in cellular iron delivery [7,8,9]. This is because extracellular ferritin can act as an iron carrier that delivers iron to cells. A single ferritin molecule can sequester up to 4500 iron atoms, potentially making it a very effective iron delivery system. Considered to be iron-poor, serum ferritin contains much less iron [7] but can still have a significant impact on iron supply. Sibille et al. examined the release of ferritin from iron-loaded Kupffer cells [8]. The results revealed that approximately 50% of the iron contained in these cells was released into the culture medium as ferritin within 24 h. When this conditioned medium was used to culture isolated hepatocytes, the released ferritin was rapidly taken up by the cells. Due to this efficient mechanism, the authors calculated that one hepatocyte could accumulate over 160,000 iron molecules per minute. This study demonstrates that exogenous ferritin can act as a highly effective iron delivery mechanism.

**Figure 2:**
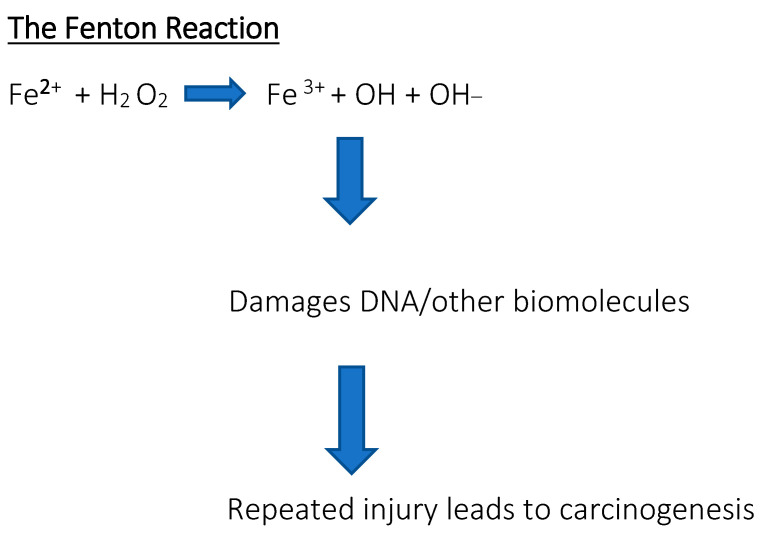
The Fenton reaction. Reaction of hydrogen peroxide with iron peroxide, iron (II), iron, and Fe^2+^, which is a method of producing a hydroxyl radical. Free iron is toxic to cells because it acts as a catalyst in the formation of free radicals from reaction from reactive oxygen species in the Fenton reaction.

Serum ferritin levels are used to assess the body’s iron content and are commonly used as a diagnostic factor in blood tests. High serum ferritin levels may indicate hereditary and acquired iron overload disorders, such as hemochromatosis or transfusion therapy, while low ferritin levels may indicate iron deficiency anemia. A positive correlation was found between the progression of the disease and the serum ferritin concentration [10,11]. Diagnostic values showed that serum ferritin is a very strong indicator of iron deficiency, much stronger than red blood cells or transferrin iron saturation [12,13,14]. Measuring the ferritin levels can provide additional information about iron storage, delivery, and inflammation, but should not be considered an absolute indicator of iron status for diagnostic or prognostic purposes. Ferritin may also be a prognostic factor in certain cancers; research results have been found for breast cancer, ovarian cancer, pancreatic cancer, and advanced non-small cell lung cancer, among others [15,16,17,18,19]. Sometimes the body is overloaded with iron, which results in the appearance of free iron, initiating reactions that produce reactive free radicals. These destroy proteins, lipids, and nucleic acids. For example, through the so-called Fenton reaction, the presence of free iron contributes to the formation of reactive oxygen species (ROS). Hydroxyl radicals are so reactive that they can damage nuclear membranes. In turn, •OH radicals lead to the formation of 8-oxo-7,8-dihydro-2′-deoxyguanosine, the presence of which results in point mutations in DNA and it is considered a good indicator of oxidative stress and a biomarker of cancer risk [20]. The interaction between free radicals and polyunsaturated fatty acids found in membranes is also dangerous (Figure 2). Lipid peroxidation causes damage to cell structures and disrupts their functions, and the accumulation of defects is associated with various diseases, including carcinogenesis [21]. When the concentration of iron exceeds the ability of proteins to bind it, the risk of developing not only cancer but also heart and nervous system diseases increases. Free iron in the body may also influence the development of bacterial pathogens that have the ability to sequester it [22].

## 3. The Function of Ferritin in the Processes of Iron Metabolism Within Cells and the Regulation of Homeostasis

**Figure 3:**
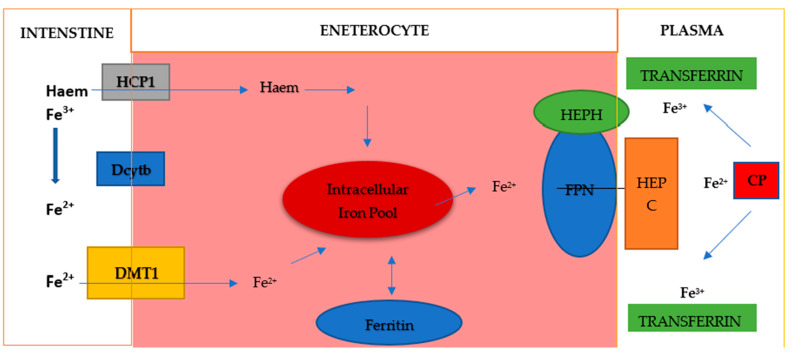
Iron metabolism. Elemental iron exists in two forms: Fe^2+^ and Fe^3+^. Fe^2+^ is the sole form absorbed from the intestine, as the intestine possesses a single ion transporter for various ionic compounds, which exclusively transports divalent ions. This transporter is known as divalent metal ion transporter-1 (DMT-1), requiring the activity of iron reductases such as duodenal cytochrome B (Dcytb). Intracellular iron can be stored in ferritin and utilized for protein biosynthesis or for the generation of reactive oxygen species (ROS) and the regulation of transcription via IRP1/IRP2. Export is facilitated by ferroportin (FPN), often aided by hephaestin (HEPH) and/or ceruloplasmin (CP) and is also inhibited by hepcidin (HEPC).

Dietary iron is mainly Fe^3+^ iron, which is reduced to Fe^2+^ iron and absorbed into cells through the coordination of reductases such as duodenal cytochrome B (DCYTB) and divalent metal transporter 1 (DMT1) on intestinal epithelial cells (Figure 3). Absorbed Fe^2+^ is excreted from intestinal epithelial cells by ferroportin on the basolateral surface, while hephaestin oxidase oxidizes it to Fe^3+^, which then binds to transferrin (TF) and is transported to various tissues and organs. Iron exported by ferroportin is also oxidized by circulating ceruloplasmin. This circulating TF-Fe^3+^ is absorbed into cells via transferrin receptor 1 (TFR1). Fe^3+^ is released in the endosome, reduced to Fe^2+^ by six-transmembrane prostate epithelial antigen 3 (STEAP3), and transported to the cytoplasm via DMT1. Fe^2+^ in the cytoplasm, which is called the labile iron pool, is metabolically active and plays a role in a variety of biological functions. Excess iron can be stored in ferritin or removed from the cell by ferroportin, where Fe^3+^ is oxidized and binds to TF in the bloodstream [23]. Excess systemic iron induces hepcidin secretion by the liver. Hepcidin binds to ferroportin and causes its degradation, reducing the outflow of iron from cells. Conversely, when systemic iron levels are low, hepcidin secretion decreases and iron efflux increases [24,25]. Iron homeostasis is mainly regulated posttranscriptionally. Iron regulatory proteins (IRP1 and IRP2) bind to iron-responsive elements (IREs) in the 5′ or 3′ untranslated region of the mRNA. In this way, appropriate protein synthesis is controlled. Proteins involved in iron import (TFR1, DMT1), ferritin (FT), and iron extraction (ferroportin) are regulated in this way [23,24]. Iron regulation primarily occurs at the posttranscriptional level when mRNA-binding proteins, iron regulatory proteins (IRP) 1 and 2, bind to the 5′ stem-loop structure of ferritin mRNA, thereby inhibiting its translation. This 5′ stem-loop structure, known as the iron-responsive element (IRE), is highly conserved and is also present in other proteins regulated by iron levels, such as the transferrin receptor. In conditions of iron deficiency, cells depend on the iron stored in ferritin and permit its degradation through proteasomal or lysosomal mechanisms to maintain intracellular iron homeostasis. During such iron-deficient states, additional ferritin translation is unwarranted, prompting cells to enforce this translation blockade via IRP1/IRP2 binding to the 5′ IRE. Under conditions of abundant iron, IRP1 typically functions as a cytosolic aconitase but adopts an open conformation that facilitates its binding to the IRE. Both FTH1 and FTL, which are encoded by distinct, highly conserved genes containing three introns and four exons, feature a highly conserved 5′ IRE [25,26]. Both IRP1 and IRP2 exhibit tissue-specific expression and regulate FTH and FTL in a context-dependent manner in various cancers.

FTH1, unlike FTL, is specifically downregulated by IRP2 in breast and prostate cancer cell lines [27,28]. The posttranscriptional regulation by miRNAs, which bind to the 3′ untranslated regions (UTRs) of mRNAs to inhibit translation or induce transcript degradation [29], is also recognized. miR335 has been demonstrated to bind directly to the FTH1 transcript, targeting it for degradation. In contrast to posttranscriptional regulation, the transcriptional regulation of ferritin genes, particularly FTH1, remains less understood. The tissue-specific activation of FTH1 gene transcription [30] is mediated by cyclic adenosine monophosphate (AMP) and hemin through a 0.1 kb B site located upstream of the transcription initiation sequence. Considerable work is still required to elucidate the transcription factors that bind to the B site and the various regulators that influence FTH1 transcription. In addition to iron, proinflammatory cytokines (TNFα and IL-1α), growth factors, hormones, oxidative stress, hypoxia, non-coding RNAs (ncRNAs), and certain proteins such as p53 also contribute to the regulation of ferritin levels in an iron-independent manner [31,32].

## 4. Hypoxia and the Tumor Microenvironment

Studies have shown that ferritin, specifically its light chain (FTL), can be regulated at the posttranscriptional level under hypoxic conditions. Alveolar macrophages have a 2.5-fold higher FTL content when the cells are exposed to hypoxia [33,34]. Hypoxia is a very common pathological feature of cancer. Tumor malignancy, EMT (epithelial–mesenchymal transition), angiogenesis, and drug resistance are activated by hypoxia. In U87 and U251 glioma cells, FTL was shown to be upregulated by hypoxia, and hypoxia-induced FTL was a positive regulator of EMT. In these cells, hypoxia-inducible factor 1-alpha (HIF 1α) directly binds to the hypoxia response element (HRE-3) region on the FTL promoter to activate its transcription [35,36]. 1-alpha (HIF 1α) is upregulated in hypoxia and is associated with the activation of tumor growth and metastasis in many cancers, including glioma, breast, and hepatocellular carcinoma [37]. The regulation of FTH1 by 1-alpha (HIF 1α) is complicated. Undoubtedly, ferritin heavy chain (FTH1) stabilizes 1-alpha (HIF 1α) by reducing PHD activity (HIF prolyl hydroxylase, which directs 1-alpha (HIF 1α) to proteasomal degradation) through Fe^2+^ deprivation. Additionally, FTH1 interacts with FIH (the HIF inhibitory factor inhibits 1-alpha (HIF 1α) transcriptional activity) and activates its hydroxylase activity, which inhibits 1-alpha (HIF 1α). This causes FTH1 to have opposite effects on the two negative regulators of 1-alpha (HIF 1α). In hypoxic primary human macrophages, FTH1 and mitochondrial ferritin expression were found to be increased, protecting these cells from iron-induced cell death [38]. In K562 tumor cells, however, hypoxia only slightly activated FTH1 translation by reducing IRE-IRP interactions. The same study showed that cobalt chloride, a hypoxia mimetic, decreased FTH1 expression by increasing IRE-IRP interactions, resulting in a translational blockade of FTH1 mRNA [39]. These studies have demonstrated the cross-regulation between hypoxia/1-alpha (HIF 1α) and ferritin and have further highlighted the differences in the use of hypoxia mimetics, such as cobalt chloride, compared to the hypoxic conditions created by maintaining 1% O_2_ gas conditions. Hypoxia in the tumor microenvironment is a complex condition that utilizes distinct mechanisms of expression of different genes, including ferritin genes, by targeting both DNA and RNA regulatory elements. While FTH1 can protect healthy cells from hypoxia damage, cancer cells, on the other hand, can use the same mechanism to their advantage in sustaining and activating tumorigenesis.

## 5. The Roles of Ferroportin and Hepcidin in Increased Iron Sequestration

Iron is essential for maintaining homeostasis, while its excess increases the risk of cancer development, mainly due to the formation of ROS. In addition, it is a key stimulus for cancer proliferation. Iron dysfunction also causes cancer-related anemia and can lead to the development of new tumors. The hepcidin–ferroportin system, which is a key pathway for regulating iron homeostasis, plays a predominant role in the whole process. Although the connections among cancer cells, iron, and the complex roles of proteins regulating iron homeostasis seem to be stable, many of the molecular mechanisms and functions of these regulators still remain unclear. Hepcidin is an important peptide regulating iron efflux from cells. Since iron is essential for cell survival, especially for highly active cells such as cancer cells, it is necessary to understand how cancer cells manipulate hepcidin expression for their own metabolic needs. Studies confirm that hepcidin expression and regulation in cancer cells show significant differences compared to its expression and regulation in non-cancerous cells [40]. These differences should be studied to develop new strategies to combat cancer cells. Manipulating hepcidin expression to deprive cancer cells of iron may prove to be a new therapy in the group of anticancer drugs. Hepcidin is an important peptide for cellular iron homeostasis. This is crucial because the level of iron charge affects the redox state in cells. Oxidative damage is a pathogenic factor associated with various diseases. Therefore, it is not surprising that hepcidin imbalance has also been associated with diseases such as diabetes, liver cirrhosis, and heart dysfunction. Although the role of hepcidin in cancer has been studied less, the data suggest that hepcidin imbalance also occurs in cancer. Hepcidin levels are increased in most cancers, with the exception of Hepatocellular carcinoma (HCC) and some brain tumors, where hepcidin levels are low. The increased hepcidin, produced by the liver, in cancers is probably the result of the accompanying inflammation. The absence of an increase in HCC is probably due to the loss of differentiation. Hepcidin imbalance in cancer is very important because it provides tumors with the iron they need for their survival [41]. The strategy that cancer cells use to achieve this is to increase cellular iron import by increasing the TFR1 levels and to decrease cellular iron export, which is accomplished through the action of hepcidin on ferroportin (FPN) [40]. Hepcidin is a hormone secreted by the liver that controls the systemic iron concentration by binding to FPN-1 on enterocytes, which reduces the export of iron into circulation [42]. The hepcidin/ferroportin axis controls systemic iron homeostasis by regulating the acquisition of iron from the duodenum and reticuloendothelial system, respective sites of iron absorption and recycling. FPN is also abundant in the kidney, where it has been implicated in tubular iron reabsorption homeostasis. The reticuloendothelial system (RES) consists mainly of monocytes and tissue macrophages. Its main roles in iron metabolism are to process iron from senescent red blood cells, and it also serves as a large storehouse of excess iron. In circulation, hepcidin will bind to FPN-1 on the basolateral surface of the cell, causing the internalization and degradation of the complex [40]. This degradation results in reduced iron export from the cell. Hepcidin expression is often increased in various tumors, as reviewed in [40]. A study in patients with pancreatic cancer showed that low FPN-1 expression in tumors combined with high hepcidin expression predicted poor survival [43]. In breast cancer tissues, FPN-1 expression was significantly reduced and indicated poorly differentiated tissue. When FPN-1 was overexpressed in breast cancer cells, there was a marked reduction in tumor growth in the mammary fat pads of mice. In a cohort of >800 women analyzed for FPN-1 and hepcidin expression in breast cancer cells, independent of other breast cancer markers, high FPN-1/low hepcidin showed a 10-year survival of >90%, whereas women with low FPN-1 /high hepcidin expression showed a 10-year survival of only 43% [44]. In prostate cancer cell lines, LNCap, DU145, and PC3, the siRNA knockdown of hepcidin resulted in a significant reduction in proliferative capacity [45]. However, this effect was shown to be a result of changes in the MAPK1 pathway and was not directly related to iron export. Therefore, the evidence suggests that cancer cells preferentially reduce their capacity to export iron [46]. Recently, there has been increasing research into the entire system consisting of the hepcidin–ferroportin axis, iron metabolism, and cancer, and potential interrelationships among them have been identified [47]. Rich and complex microenvironments that include stroma, endothelial cells, and inflammatory cells, including macrophages and tumor cells have also been identified. Extracellular and intracellular iron may be a major regulator of hepcidin expression in tumors. Cancer cells must retain iron to proliferate and take up more iron for growth. The suppression of hepcidin secreted by the liver seems possible, because duodenal enterocytes transfer iron to plasma, which increases the total body iron content [48,49]. Hepcidin expression levels also depend on divergent cell types and states. Previous studies have identified several factors that regulate hepcidin mRNA expression. For example, anemia and hypoxia lead to decreased hepcidin levels [50]. As malignant tumors outgrow their blood supply, tumor cells are often deprived of oxygen. This may explain the suppression of hepcidin in some tumors. However, hepcidin mRNA is absent in cell lines grown under optimized and normoxic conditions, such as astrocytoma cell lines [51]. This may be due to the lack of several key factors that induce intratumoral hepcidin expression. Furthermore, hypoxia is a key regulator of various genes in growing tumors, which may affect hepcidin expression [52,53]. Although the results induced by hypoxia are inconsistent, the function of this regulator in cancer and tumor cells should be intensively studied.

The iron status in the microenvironment is mainly reflected by circulating iron transferrin and iron released from cells in the microenvironment. It has been proven that cells in the microenvironment can supply iron to cancer cells. Tumor-associated macrophages, a specific type of macrophage, secrete cytokines in a similar manner to M2-polarized macrophages. Recent studies have shown that M2-polarized macrophages exhibits an iron phenotype that is significantly different from inflammatory M1 macrophages [54,55], and the expression of FPN and the downregulation of ferritin and heme oxygenase have been detected in M2 macrophages. All these changes can cause iron release [55]. From these findings, we can conclude that iron is involved in the cross-talk between cancer cells and their environment.

## 6. Ferritin as a Marker of Acute and Chronic Inflammation

Serum ferritin is classified as a marker of acute and chronic inflammation and is elevated in many inflammatory conditions, including rheumatoid arthritis, systemic lupus erythematosus, chronic kidney disease, COVID-19, acute infection, thyroiditis, and others [56,57]. Interestingly, increased levels of ferritin have also been detected in the synovial fluid and cells of patients with rheumatoid arthritis. Additionally, it has been proven that the level of ferritin correlates with the severity of the disease [10,11]. Ferritin has been proven to be a marker of inflammation and the activation of M1 macrophages. Hyperferritinemia (serum ferritin concentration of ≥500 ng/mL) is a potential marker which can be used to differentiate hospitalized patients with confirmed influenza A infection, associated with serious complications such as respiratory failure, among others [58]. However, a significant increase in the serum ferritin concentration may indicate the activation of the monocyte–macrophage system, which is involved in the activation of part of the inflammatory cytokine storm. It should also be noted that patients with severe COVID-19 often die due to the resulting cytokine storm, which is an excessive, uncontrolled inflammatory response. Serum inflammatory biomarkers, such as interleukin-6, coagulation markers, and ferritin, indicate a potential inflammatory storm in most patients with severe COVID-19. However, the study did not take into account what type of diabetes was being referred to. It is of interest whether ferritin is a mediator or a consequence of inflammation. It seems highly likely that H-ferritin modulates the response of macrophages to immune stimuli and plays an important role in protection against iron-induced oxidative stress [59]. The overexpression of H-ferritin in macrophages leads to their polarization depending on the cytokines that are present in their environment [60]. However, many previous studies have described the proinflammatory and immunosuppressive roles of ferritin, which may depend not only on the context and the different signaling pathways activated, but also on the role and location of the ferritin molecules involved, the H/L ratio in multimers, and the potential role of ferritin in these inflammatory pathways. Ferritin’s function as a source or store of iron appears to be crucial, and its regulation may explain various observations. In kidney disease, elevated ferritin levels have been shown to be associated with increased mortality in three study regions covering Europe, Japan, and the United States, despite different median ferritin levels across regions. However, the use of ferritin as a biomarker here is limited due to intravenous iron dosing, inflammation, anemia treatment strategies, or diet, which may vary by region [61]. Dialysis patients often take iron supplements due to iron loss during the dialysis process, opening up a discussion about the safety of iron supplementation. It is also unclear to what extent ferritin and iron influence the etiology of this disease. Therefore, further research is warranted here to shed light on this issue and improve patient care.

Both low and high levels of ferritin in the blood may indicate serious illness (Table 1 and Table 2).

**Figure 4:**
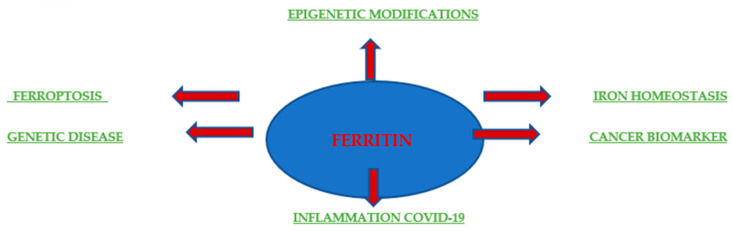
Ferritin in health and disease. Schematic depicting the involvement of ferritin in disease settings and biomedical applications.

Originally, ferritin was known for its role in iron storage and used to indicate the total amount of iron in the body. Additionally, elevated ferritin levels often indicate anemia or chronic disease (Figure 4). The pathophysiological function of ferritin remains unclear, but elevated serum ferritin levels are correlated with the processes of inflammation, infection, and liver disease, demonstrating the potential of ferritin as a prognostic factor and clinical indicator. Serum ferritin consists mainly of L chains under normal physiological conditions, whereas heavy ferritin and the ratio of H to L ferritins are increased in many malignant conditions. In addition, an increase in ferritin correlates with the stage of disease advancement and with local release in the tumor microenvironment, as was the case with breast cancer [62]. A few studies have also assessed the prognostic value of ferritin as a predictor of relapse and progression in patients. Research conducted by Ahmed A. et al. in 2012 at the University of Pennsylvania provided a molecular mechanism by which the presence of ferritin in the tumor microenvironment directly stimulates tumorigenesis. It is becoming increasingly clear that ferritin plays a multifunctional role in human biology. It is involved in the proliferation [63,64], angiogenesis [64], and provision of iron [65,66]. The H subunit is responsible for iron supply, while the L subunit is associated with proliferation and angiogenesis. For example, the overexpression of L-ferritin, but not H-ferritin, increased cell proliferation in HeLa cells without affecting the intracellular iron levels [67]. Coffman et al. drew similar conclusions; they showed that L-subunit-rich ferritin can bind to high-molecular-weight kininogen and block its anti-angiogenic effect on endothelial cells. However, the mechanism by which ferritin stimulates HeLa cell growth or blocks the antiangiogenic effects of high-molecular-weight kininogen is still undiscovered and is believed to be independent of iron. The study by Ahmed A. et al. provided evidence that L-ferritin-rich complexes can directly interact with breast cancer cells and stimulate proliferation independently of iron.

## 7. Ferritin in Cancer

Although the mechanism by which ferritin stimulates breast cancer cells is still unknown, evidence has shown that it is not due to the iron supply and is not dependent on the iron content of ferritin. It has also been proven that the putative L-ferritin receptor Scara5 is not sufficient for proliferative effects, as originally speculated [7], increasing the possibility of the involvement of another L-ferritin receptor. Ferritin is the main intracellular iron storage protein and is abundant in circulation. It has also been proven that in breast cancer patients, ferritin is detected in higher concentrations both in serum and in tumor lysates, and its increase correlates with poor clinical outcomes [61]. Research conducted at the University of Pennsylvania comprehensively examined the distribution of ferritin in normal and malignant breast tissue at various stages of tumor development. Decreased ferritin expression in tumor cells but an increased infiltration of ferritin-rich CD68-positive macrophages was observed with increasing tumor histological grades [68,69]. Studies have been conducted on the possible functional significance of extracellular ferritin in a breast cancer cell culture model. Ferritin stimulated the proliferation of the MCF7 and T47D epithelial breast cancer cell lines. Moreover, this proliferative effect was independent of the iron content of ferritin and did not increase the intracellular iron levels in cancer cells, indicating a novel, iron-independent function of this protein. Collectively, these findings suggest that ferritin released by macrophage infiltration in breast tumors may represent an inflammatory effector mechanism, resulting in ferritin directly stimulating tumorigenesis [70]. As is already known, the functions of ferritin are traditionally associated with intracellular iron storage, but recently, additional roles have been discovered and studied [68]. For example, H-ferritin can act as a molecule that delivers iron to the brain, and more specifically to oligodendrocytes, through endocytosis via the mouse H-ferritin Tim-2 receptor [22,71]. On the other hand, complexes rich in L-ferritin can bind to high-molecular-weight kininogen and block its antiangiogenic effect on endothelial cells independently of iron [72,73].

In contrast to breast cancer, patients with colorectal cancer exhibit lower ferritin levels compared to healthy controls [74]. Notably, under hypoxic conditions, which are characteristic of many solid tumors [75], ferritin expression is upregulated. This phenomenon has been examined in the alveolar cells of lung tumors, where ferritin expression increases under hypoxic conditions, independent of variations in iron levels [76]. Recent in vitro studies indicate that elevated ferritin levels, particularly the H subunit, observed in ovarian cancer, may contribute to chemoresistance to therapies. This is substantiated by the fact that many anticancer drugs function by generating reactive oxygen species (ROS). Increased FTH levels may diminish ROS levels, thereby reducing drug efficacy. The authors have proposed FTH inhibition as a potential strategy to alleviate chemoresistance in ovarian cancer [77]. The role of ferritin in this pathology remains inadequately explored and necessitates further investigation. Interestingly, numerous research studies have established a connection between iron metabolism, tumor biology, and immune surveillance. Iron stimulates the production of reactive oxygen species (ROS), which may lead to iron-induced cell death or malignant transformation, necessitating elevated iron levels for cell proliferation. These processes are regulated by inflammatory cytokines, which in turn influence the transcription of hepcidin—a crucial component of iron metabolism—resulting in the degradation of ferroportin and the sequestration of iron in ferritin [78]. Conversely, ferroptosis represents a significant mechanism that promotes cell death [79]. Tsoi et al. proposed the use of ferroptosis inducers as a complementary approach to conventional treatments that specifically address the plasticity of dedifferentiation associated with melanoma cells in cases of recurrent innate and acquired resistance [80]. Recent research over the past decade has yielded insights into the application of ferritin H nanocarriers for targeted drug delivery to specific cells. This is attributed to the ability of FTH to bind selectively to the transferrin receptor [81]. Notably, it has been demonstrated that only FTH, and not FTL, is directed towards cancer cells [82]. The effectiveness of this innovative approach has already been validated in murine models of gastric cancer [83] and colon cancer [84] for the delivery of the anticancer agent doxorubicin. This is particularly noteworthy as recent findings indicate that the FTH nanocarrier system can traverse the blood–brain barrier (BBB) and effectively eliminate glioma cells. This phenomenon is linked to the high expression of TfR, the entry point for the FTH nanocarrier, in both BBB endothelial cells and glioma cells. Furthermore, the authors discovered that this nanocarrier exits the BBB via the endosomal compartment, without accumulating in healthy brain tissue [82].

## 8. Ferritin as a Potential Cancer Biomarker

Based on the above, it is justified to examine the serum ferritin concentration, which is elevated in many human cancers, including breast cancer [85,86]. The functional significance of this increase has been consistently ignored due to the dominant paradigm regarding ferritin as a protein whose primary role is to store iron and a nonspecific acute phase reactant. The available data may be of interest, indicating that this increase correlates with the stage of disease advancement in breast cancer patients [86,87] and that increased serum ferritin levels may be related to local release in the breast tumor microenvironment [88,89]. Several studies have assessed the prognostic value of serum ferritin, and present ferritin as a predictor of recurrence and progression in breast cancer patients [90,91]. In one study, serum ferritin showed prognostic value independent from the other inflammatory biomarkers, suggesting functional significance [90]. Additionally, breast tumor lysates also show increased levels of L-ferritin, the dominant subunit in serum [91]; this increase correlates with an advanced histological grade and shorter survival [92,93,94,95,96]. Histological examinations of ferritin distribution in the breast showed weak staining of the ductal cells in benign breast tissue, moderate staining in the breast cancer cells, and strong staining in the tumor stroma [93,95].

It has been proven that an increase in ferritin concentration in breast tumors, as well as in the serum of breast cancer patients, correlates with poor clinical outcomes [45,74,86,93].

Serum ferritin, which is often overlooked as an associated protein, is elevated during acute and chronic inflammation, probably as a result of the activation of transcription factor NF-kB [97]. In addition, chronic use of anti-inflammatory drugs significantly reduces serum ferritin levels in patients with inflammatory diseases [98]. Moreover, elevated serum ferritin levels have prognostic and predictive value in patients with advanced breast cancer, regardless of the inflammatory biomarker C-reactive protein, suggesting that ferritin may play a functional role [86]. Cancer-related elevated serum ferritin levels may represent a functional link between inflammation and tumorigenesis. Therefore, increased ferritin during inflammation and infection requires further research.

There is plenty of evidence suggesting that cancer-related increases in serum ferritin levels are partly due to local secretion within the tumor. In breast cancer patients with increased serum ferritin levels, surgical resection of the tumors resulted in a decrease in the serum ferritin levels by approximately 50%, indicating a relationship between tumor mass and increased serum ferritin levels [99]. An analysis of intraductal fluid in breast cancer patients also showed a five-fold increase in ferritin levels compared to healthy controls, suggesting a local release of ferritin in the breast tumor microenvironment [100]. Elevated serum ferritin levels have been noted in several types of cancer. However, little is known about the relationship between ferritin and glioma. Glioma is the most common type of primary brain tumor in humans. It turns out that glioblastoma multiforme (GBM) patients show significantly elevated preoperative ferritin levels (i.e., ferritinemia) within the serum ferritin reference range and marked ferritin immunoreactivity in resected tumor tissue [101]. An indirect relationship was observed between ferritin synthesis in glioma tissue and altered ferritin levels, which limits the clinical value of ferritin as a glioma tumor marker [55]. It has also been demonstrated that GBM-derived glioma cells release ferritin in vitro, which has an apoptosis-stimulating effect. Although the pathophysiological context in which tumor-derived ferritin induces apoptosis remains to be determined, recent findings elucidate a clear growth-regulatory role of these ferritin species in tumor biology [102,103].

Furthermore, the increased immunoreactivity of resected GBM tissue for the FTL subunit is responsible for the synthesis of the FTL-type isoform, which corresponds well with the reported expression of FTL-type isoferritins in cultured glioblastoma multiforme-derived cells and similar findings in glioblastoma stem cells [104]. Although the pathophysiological significance of this new finding is elusive, previous studies have shown that iron-mediated oxidative stress and lipid peroxidation play key roles in ferritin-mediated apoptosis [105]. Of note, increased iron requirements have been demonstrated in glioblastoma stem cells, where increased ferritin expression presumably provides stable intracellular iron buffering [105].

A similar situation may also apply to benign brain tumors, such as meningioma, which, as has been proven, may also be accompanied by increased serum ferritin levels [106].

Ferritin was detected immunocytochemically in the cytoplasm of tumor cells in resected glioblastoma. Thus, it seems that CSF ferritin is produced by glioblastoma cells, and its biological significance requires further investigation [107].

Since the exact reason for the involvement of ferritin in pancreatic carcinogenesis has not yet been fully established, previous studies have shown that SF can originate from hepatocytes, macrophages, and microglia and contain both FTH1 and FTL25 ferritin subunits. It has also been reported that SF in malignant histiocytosis is mainly composed of FTH1, whereas SF in breast cancer patients is strongly correlated with FTL26–28 [6,18]. Recent research also shown a possible link between ferritin and pancreatic cancer, although these findings are not yet definitive. Pancreatic cancer is one of the deadliest diseases and lacks an early diagnostic marker. Strong associations between ferritin and pancreatic cancer have been noted [18]. It turns out that high SF levels may indicate a risk of pancreatic cancer, which suggests ferritin as a new tumor marker that can be used in the diagnosis of pancreatic cancer [101]. Ferritin assessments may provide a potential marker to predict individuals at increased risk during pancreatic cancer screening and may represent a new therapeutic strategy for pancreatic cancer. Ferritin is an independent predictor of mortality in patients with pancreatic cancer. It has also been shown that a high SF level at the time of pancreatic cancer diagnosis indicates a poor prognosis for the patient [18].

Increased exhaled ferritin has been observed in patients with non-small cell lung cancer, but it has not been established whether it can be used as a biomarker [106]. Kukulj S. et al. analyzed the influence of iron parameters and inflammation on the overall survival of patients with non-small cell lung cancer (NSCLC). In addition, the expression of transferrin receptor 1 (TfR1) and ferritin in tumor tissue, tumor stroma, and normal lung tissue was analyzed. Iron metabolism and inflammation parameters were determined through automated laboratory measurements at the time of diagnosis. The expressions of TfR1 and ferritin were determined using immunohistochemical methods. About 50% of patients survived for only 12 months. At the time of diagnosis, more than half of the patients had anemia and significantly elevated serum ferritin levels. The iron content in the serum ferritin (ICF) was below the reference values in 90% of the patients. Furthermore, the ICF showed a positive correlation with the iron metabolism parameters and survival, but a negative correlation with the serum ferritin and ESR. The expressions of TfR1 and ferritin in the tumor cells were observed in 88% or 62% of the patients, respectively. The tumor stroma was TfR1-negative and sporadically ferritin-positive. Ferritin expression in the tumor tissue showed a negative correlation with the serum iron and hematocrit (Ht) and a positive correlation with the ferritin, ESR, alpha-1-globulin, and alpha-2-globulin. A positive correlation was found between the tumor TfR1 expression and the alpha-globulin. No correlation was observed between TfR1/ferritin expression in the tumor tissue and the ICF or survival. Therefore, we conclude that elevated serum ferritin levels in the sera of patients with NSCLC are a result of inflammation and oxidative stress, not iron overload. Higher ferritin concentrations in tumor tissue may be a consequence of iron deficiency or local toxicity induced by environmental factors [18]. Studies conducted in 2017 also showed that iron plays a role in the tumor microenvironment and in metastasis. Cancer cells show an increased dependence on iron compared to their normal counterparts—a phenomenon called iron addiction. A higher intake of heme iron has shown a tendency toward a positive association with the risk of cancer [104]. The World Health Organization has recommended that the SF concentration is the best indicator of iron deficiency. SF is highly expressed in tumor tissues and the serum of patients with NSCLC [98,99].

Recent studies have also shown that high levels of ferritin were observed in lung cancer, and its concentration can be considered helpful in the diagnosis of malignant tumors [106]. A study assessed the diagnostic value of ferritin in combination with other biomarkers, including CA125, carcinoembryonic antigen (CEA), Neuron-specific enolase (NSE), and CYFRA 21–1, in the diagnosis of early lung cancer in older people [106]. The serum ferritin levels were shown to be significantly higher in patients with lung cancer compared to patients with benign lung disease and healthy participants [106]. Increased serum ferritin levels have been observed in both NSCLC and Small Cell Lung Cancer (SCLC) patients [98,99].

Changes in ferritin expression, independent of iron metabolism, are associated with many types of cancer. These cancers include renal cell carcinoma (RCC) [21,107]. RCC represents the sixth most frequently diagnosed cancer in men and the tenth in women [108]. Due to its high incidence and mortality, the detection of RCC at an early stage is crucial for patient survival. Mason and Taylor were the first to demonstrate the presence of ferritin in renal tubules using immunohistochemical staining techniques [17,109]. This was further demonstrated by Mufti et al. in their case report [8,110]. Fleming et al. identified ferritin in 54% of neoplastic renal tissue using immunohistochemical staining [9,111]. Partin et al. reported more intense iron staining in tumors with intramural hemorrhage or necrosis than in tumors lacking these features. Based on these findings, they postulated increased serum ferritin levels, which may represent a combination of necrotic leakage from the tumor and increased hematopoietic synthesis or increased release from the tumor [111]. Other possible explanations for elevated serum ferritin levels include an acute phase reaction, increased production, or decreased clearance. In a study by Singh KJ et al., it was observed that higher serum ferritin levels were observed with an increasing tumor stage, grade, size, and volume [112]. The investigators hypothesized that there is a greater likelihood of necrosis and hemorrhage in tumors at a higher stage, grade, size, and volume compared to tumors at a low stage, size, grade, and volume. Tumor size was more strongly correlated with the serum ferritin levels than the tumor volume, which is different from what was reported by Partin et al., who found that tumor volume correlated more strongly than tumor size. The investigators concluded that serum ferritin levels correlated well with tumor size, grade, grade, and volume. Further studies are needed to determine its exact role in RCC [113].

It has been proven that the expression of FtH (ferritin heavy chain) in the kidneys is an effective biomarker of RCC, where increased FtH concentrations are closely associated with worse treatment outcomes [114]. FtH may increase the antioxidant potential and thus promote the survival of cancer cells. On the other hand, emerging data show that FtH can inhibit tumor growth by interacting with survivin [115]. Survivin is a regulatory protein that controls apoptosis, cell division, and metastasis and is often overexpressed in cancer cells. Studies have shown that the exposure of cancer cells to a recombinant peptide containing the FtH domain interacting with survivin leads to a reduction in the growth and viability of cancer cells. Although this finding suggests additional functions for FtH, further studies need to be conducted to fully understand the implications of ferritin during tumorigenesis. Serum ferritin has also been used as a tumor marker in RCC and shows a strong correlation with the RCC stage and renal tumor volume [116,117]. The use of ferritin as a marker for RCC may provide an accurate diagnosis and enable the development of an effective treatment regimen.

## 9. Conclusions

Ferritin has been the subject of continuous study for over 85 years, and its role as the principal iron storage protein within cells is well established. The intracellular functions of ferritin are largely characterized. Additionally, ferritin is a multifunctional protein involved in proliferation, angiogenesis, immunosuppression, and iron delivery. In the context of cancer, elevated levels of ferritin are observed in the serum of cancer patients, with higher concentrations correlating with aggressive disease and unfavorable clinical outcomes. Furthermore, ferritin is highly expressed in tumor-associated macrophages, which are pivotal in tumor progression and resistance to therapy. These characteristics render ferritin a compelling target for cancer treatment, as its reduction can disrupt the supportive tumor microenvironment, induce tumor cell death, and enhance sensitivity to chemotherapy. In conclusion, in light of the available data, it can be speculated that increased serum ferritin concentrations in cancer patients may correlate with the stage of cancer advancement and may constitute an effective prognostic factor and tumor biomarker [89,118]. Moreover, ferritin is used in clinical therapy. For example, due to its unique structural advantages, it is used as a carrier of drugs targeting cancer cells [119]. The current state of the art of the scientific interrogation into the versatility of ferritin is likely just the tip of the iceberg when it comes to understanding the role of this unique protein in cancer and its dynamic roles.

## Figures and Tables

**Table 1:** Serum ferritin concentrations in normal males and females of various ages.

	Serum Ferritin
Babies < 1 years old	12.0–327.0 ng/mL
Children 1 to 3 years old	6.0–67.0 ng/mL
Children 3 to 6 years old	4.0–67.0 ng/mL
Women 6 to 12 years old	7.0–84.0 ng/mL
Men 6 to 12 years old	14.0–124.0 ng/mL
Women 12 to 17 years old	13.0–68.0 ng/mL
Men 12 to 17 years old	14.0–152.0 ng/mL
Women > 17 years old	13.0–150.0 ng/mL
Men > 17 years old	30.0–400.0 ng/mL

**Table 2:** Interpretations of high and low serum ferritin concentrations.

Low Serum Ferritin	High Serum Ferritin
Insufficient iron intake from food Impaired iron absorption in the intestine Heavy menstrual bleeding Rectal bleeding Massive internal bleeding	Hemochromatosis Iron overload—massive blood transfusions, ineffective red blood cell production, hemodialysis Liver diseases—cirrhosis and liver cancer Long-term inflammation during chronic diseases Infections Leukemias and lymphomas Pancreatic cancer Lung cancer Neuroblastoma Hyperthyroidism Megaloblastic anemia Hemolytic anemia Thalassemia Porphyria Alcoholism
